# Predictors of Seeking Health Information and Mental Health Support in U.S. Prisons: A Study Using 2014 PIAAC Data

**DOI:** 10.1093/geroni/igaa057.1082

**Published:** 2020-12-16

**Authors:** Candidus Nwakasi, Darlingtina Esiaka, Janardan Subedi

**Affiliations:** 1 University of Southern Indiana, Evansville, Indiana, United States; 2 Union College, Union College, New York, United States; 3 Miami University, Miami University, Ohio, United States

## Abstract

Being in prison increases the vulnerability to poor health, especially mental illnesses. This is evident in the documented health disparities between prison inmates and the general population. For example, suicide rates among inmates are higher than in the general population. There is an urgent need to understand how inmates experience mental well-being. This is important as some inmates serve long/life sentences and some will need to successfully re-integrate into the society. Although they have a constitutional right to health care access through the Eight Amendment, little is known of the health information and mental health support seeking patterns among inmates. The current study examined factors associated with the amount of health information accessed, and participation in mental health support groups in US prisons. Data (N= 645) from the Program for the International Assessment of Adult Competencies (2014) were analyzed using linear and logistic regressions. Sample weights were applied in the analyses. Results show statistically significant relationships between amount of health information acquired and age (66 years and above), race, health-status, readiness to learn, literacy skill, and numeracy skill. Social trust moderated the effect of education on the odds of participating in mental health support groups. Also, gender, work duration, attending substance abuse support and life skills groups were significant predictors. Our study may provide insight for stakeholders (e.g., policymakers, clinicians, social workers, and wardens, etc.) working in partnership to deliver a more tailored health interventions for inmates, by highlighting key contextual issues predicting mental health and well-being within prison settings.

